# Daptomycin > 6 mg/kg/day as salvage therapy in patients with complex bone and joint infection: cohort study in a regional reference center

**DOI:** 10.1186/s12879-016-1420-7

**Published:** 2016-02-17

**Authors:** Sandrine Roux, Florent Valour, Judith Karsenty, Marie-Claude Gagnieu, Thomas Perpoint, Sébastien Lustig, Florence Ader, Benoit Martha, Frédéric Laurent, Christian Chidiac, Tristan Ferry, on behalf of the Lyon BJI Study group

**Affiliations:** Department of Infectious Diseases, Hospices Civils de Lyon - Hôpital de la Croix-Rousse, 103, Grande-Rue de la Croix-Rousse, 69317 Lyon, cedex 04 France; Claude Bernard Lyon 1 University, Lyon, France; International Center for Research in Infectiology, CIRI, INSERM U1111, CNRS UMR5308, ENS de Lyon, UCBL1 Lyon, France; Department of Infectious Diseases, William Morey Hospital, Chalon-sur-Saône, France; Specialized Unit of Pharmacology, Hospices Civils de Lyon, Lyon, France; Department of Orthopaedic Surgery, Hospices Civils de Lyon, Lyon, France; Laboratory of Bacteriology, French National Reference Center for Staphylococci, Hospices Civils de Lyon, Lyon, France

**Keywords:** Daptomycin, Bone and joint infection, Eosinophilic pneumonia

## Abstract

**Background:**

Even if daptomycin does not have approval for the treatment of bone and joint infections (BJI), the Infectious Diseases Society of America guidelines propose this antibiotic as alternative therapy for prosthetic joint infection. The recommended dose is 6 mg/kg/d, whereas recent data support the use of higher doses in these patients.

**Methods:**

We performed a cohort study including consecutive patients that have received daptomycin >6 mg/kg/d for complex BJI between 2011 and 2013 in a French regional reference center. Factors associated with treatment failure were determined on univariate Cox analysis and Kaplan-Meier curves.

**Results:**

Forty-three patients (age, 61 ± 17 years) received a mean dose of 8 ± 0.9 mg/kg/d daptomycin, for a mean 81 ± 59 days (range, 6–303 days). Most had chronic (*n* = 37, 86 %) implant-associated (*n* = 37, 86 %) BJI caused by coagulase-negative staphylococci (*n* = 32, 74 %). A severe adverse event (SAE) occurred in 6 patients (14 %), including 2 cases of eosinophilic pneumonia, concomitant with daptomycin C_min_ >24 mg/L. Outcome was favorable in 30 (77 %) of the 39 clinically assessable patients. Predictors for treatment failure were age, non-optimal surgery and daptomycin withdrawal for SAE.

**Conclusions:**

Prolonged high-dose daptomycin therapy was effective in patients with complex BJI. However, optimal surgery remains the cornerstone of medico-surgical strategy; and a higher incidence of eosinophilic pneumonia than expected was recorded.

## Background

Bone and joint infections (BJI) include very heterogeneous clinical entities, with different therapeutic strategies and prognoses. Some, such as uncomplicated childhood osteomyelitis, are easy to treat, as short-course antimicrobial therapy without surgery is classically associated with excellent prognosis. In contrast, in some situations such as chronic implant-associated BJI, the pathogen is difficult to eradicate, leading to high rates of relapse and morbidity despite aggressive surgical strategy and prolonged intravenous antibiotic therapy. In such cases, team-work in tertiary care centers is required to determine optimal surgical management so as to limit treatment failure, motor disability and risk of amputation. The choice of antimicrobial therapy is also challenging, requiring consideration of: (i) the problem of bone diffusion [1]; (ii) the necessity of using antimicrobials active against bacterial biofilms [2]; (iii) the growing incidence of antibiotic resistance; and (iv) the high risk of severe adverse events (SAE) in response to first-line antimicrobials in these patients [3]. Consequently, off-label use of recently developed antimicrobials, such as daptomycin, is frequently required as salvage therapy in complex BJI.

Daptomycin is a cyclic lipopeptide with rapid bactericidal action against Gram-positive bacteria, and is approved for complicated skin and skin-structure infection (4 mg/kg/day intravenously) and *Staphylococcus aureus* bacteraemia and/or right-side endocarditis (6 mg/kg/d). Beyond these FDA and European-approved indications, daptomycin is increasingly used in BJI, as: (i) Gram-positive cocci are the most frequent pathogens in BJI; (ii) staphylococci exhibit growing resistance to beta-lactams and glycopeptides; (iii) a randomized controlled trial supported the use of daptomycin in these patients [4]. Consequently, daptomycin has been proposed as an alternative therapeutic option in patients with staphylococcus or enterococcus prosthetic joint infection in the recent Infectious Diseases Society of America (IDSA) guidelines (6 mg/kg/d) [5]. As bone penetration of daptomycin is limited, some authors proposed higher doses in BJI: i.e., 8 mg/kg/d [6]. This attitude is supported by the retrospective post-marketing Eu-CORE® study, but prospective data are lacking, especially in patients at high risk of relapse receiving prolonged daptomycin therapy [7].

In this context, the present cohort study aimed to assess the safety and efficacy of prolonged high-dose (>6 mg/kg/d) daptomycin salvage therapy in patients with complex BJI, focusing on daptomycin-related SAEs such as rhabdomyolysis or eosinophilic pneumonia, and to determine risk-factors for treatment failure.

## Methods

We performed a cohort study including consecutive patients that have received daptomycin >6 mg/kg/d as alternative therapy for complex BJI from January 2011 to July 2013 in a French regional reference center. Patients with creatinine clearance <30 mL/min were excluded. Diagnosis of BJI was based upon clinical and/or imaging evidence, and culture of microorganisms from synovial fluid or periprosthetic tissue (more than two specimens for bacteria from skin flora: e.g., coagulase-negative *staphylococci*, *Corynebacterium* spp, *Propionibacterium acnes*). Infection was classified as “chronic” when there was a delay of one month between the bone contamination (previous surgery) and diagnosis. In line with the definition given by the Health-care Supply Office (*Direction Générale de l’Offre de Soins, DGOS*) of the French Health Ministry (*Ministère des Affaires Sociales et de la Santé*), BJI was considered complex in the following circumstances: (i) relapsing BJI; and/or (ii) intolerance to first-line antimicrobial therapy; and/or (iii) requirement of large bone resection and/or reconstruction; and/or (iv) multi-drug resistant pathogen limiting therapeutic opportunities, such as a glycopeptide-resistant Gram-positive isolate.

Susceptibility to glycopeptides and daptomycin was assessed in methicillin-resistant staphylococci based on the criteria of the European Committee on Antimicrobial Susceptibility Testing (EUCAST: Breakpoint tables for interpretation of minimum inhibitory concentrations [MICs] and zone diameters, version 4.0), if the strain was available at the time of daptomycin prescription. Notably, daptomycin MICs were determined by the E-test® method. In patients with implant-related infection, surgical treatment was considered optimal if based on French and IDSA guidelines: i.e., debridement-lavage and implant retention followed by antimicrobial therapy in acute infection, and implant removal (with or without reimplantation) followed by antimicrobial therapy in chronic infection. In patients with non-implant-related BJI, surgery was considered optimal when large debridement including bone resection was performed when required. Daptomycin was administered intravenously once daily by 30-min infusion.

All patients were scheduled for inclusion in the “safety analysis”. Only patients for whom at least two cultures were positive for Gram-positive micro-organisms from deep surgical specimens or joint-aspiration fluid were to be included in the “efficacy analysis”. The use of high doses of daptomycin entailed particular focus on daptomycin-related adverse events (AE), which were systematically collected and assessed on the Common Terminology Criteria for Adverse Events (CTCAE: version 3.0, published 9 August 2006). AEs were classified as mild (CTCAE stage 1), moderate (stage 2) or SAE (stages 3, 4 and 5). Standard biological testing, including blood creatine kinase (CK), was performed at least once a week. After October 2012, daptomycin trough plasma concentration (C_min_) was determined monthly for all patients, and additionally in case of AEs directly implicating daptomycin (i.e., at onset of elevated blood CK or of eosinophilic pneumonia), to detect any patients with daptomycin trough levels (C_min_) over 24 mg/L. C_min_ was determined 24 h after daptomycin injection (just before reinjection, if performed) using a validated HPLC process developed in the laboratory and a diode array UV detector. C_min_ > 24 mg/L was considered an overdose, as the risk of high CPK levels (with or without symptoms of myopathy) is greater above this threshold [8].

Treatment failure was defined as worsening or new clinical signs related to persistent and/or new infection at the BJI site. Treatment failure implicating daptomycin included relapse with the same Gram-positive strain, and super-infection due to another Gram-positive strain. Super-infection due to Gram-negative strains or fungi was not considered as treatment failure implicating daptomycin. Determinants of treatment failure were explored by univariate log-rank test and unadjusted Cox analysis. Statistical analysis used SPSS 17.0 software (SPSS Inc, Chicago, IL, USA). The research was conducted in accordance with the Declaration of Helsinki and national and institutional standards. The study received the approval of the French South-East II and III ethics committee with the reference number CAL2011-021 and n°QH 20/2014. In accordance with French legislation, written informed consent was not required for any part of the study, as (i) daptomycin was used as salvage therapy in the usual care of patients addressed to our regional reference center for complex BJI; (ii) patients were informed orally and by letter that data were being collected using a national database established by the DGOS (in line with ruling n°2012–220 of the French national data protection commission: *Commission Nationale de l’Informatique et des Liberté*). The study was registered to ISRCTN (number ISRCTN14244698).

## Results

### Baseline patient characteristics

During the study period, 43 complex BJIs were treated by daptomycin (>6 mg/kg/d) as salvage therapy in 42 patients. In 40 cases (93 %), osteomyelitis was diagnosed based on clinical signs: fistula (*n* = 26, 60 %), pain (*n* = 21, 49 %), inflammatory aspect (*n* = 17, 40 %), fever (*n* = 9, 21 %), abscess (*n* = 5, 12 %), osteoarthritis (*n* = 2, 5 %), or sepsis (*n* = 1, 2 %). Three patients (7 %) were asymptomatic: in 2 cases, daptomycin was prescribed for a super-infection documented on an explanted orthopaedic device; in the third, osteomyelitis was diagnosed from radiologic abnormalities.

Criteria for complexity were: (i) relapsing BJI in 27 patients (62 %); (ii) intolerance to first-line antimicrobial therapy in 42 (98 %); (iii) requirement of large bone resection and/or reconstruction in 37 (86 %); and (iv) infection with a glycopeptide-resistant Gram-positive strain in 22 (51 %). Glycopeptides were the most common prior treatment (*n* = 37, 86 %). The most common reason for discontinuing prior antibiotic therapy was the occurrence of a side-effect (*n* = 23, 53 %), including allergic reaction (*n* = 17, 40 %) and/or renal failure related to vancomycin overdose (*n* = 7, 16 %). Baseline characteristics are summarized in Table 1.

**Table 1:** Demographic characteristics of 43 patients who received daptomycin-based antimicrobial therapy

Characteristic	(*n*, %)
Gender	
Female	18 (42)
Male	25 (58)
Mean age, years (range)	61 (18–88)
Age groups	
< 65 years	25 (58)
≥ 65–< 75 years	8 (19)
≥ 75 years	10 (23)
Mean body weight, kg (range)	75 (32–110)
Mean body mass index (range)	27 (14–43)
BMI ≥ 25, < 30	9 (21)
BMI ≥ 30	14 (33)
Ethnicity (caucasian)	42 (98)
Active smoking	10 (23)
Underlying diseases	
Hypertension	17 (40)
Diabetes mellitus	5 (12)
Cardiovascular disease	13 (30)
Cancer	6 (14)
Creatinine clearance (MDRD) 30–60 mL/min	3 (7)
Immunosuppression	4 (9)
Mean ASA score (range)	2.2 (1–4)
Past episode of BJI on the same site	27 (63)
Anatomical site of infection	
Knee	12 (28)
Hip	9 (21)
Lower limb	9 (21)
Rachis	8 (19)
Upper limb	2 (5)
Other	1 (2)
Chronic osteomyelitis	37 (86)
Physiopathology of osteomyelitis	
Post-operative	41 (95)
Hematogenous infection	2 (5)
Pressure ulcer	2 (5)
Orthopedic device	
Prosthesis	23 (53)
Osteosynthesis device	14 (33)
None	6 (14)
Previous exposition to antimicrobials for the index BJI before daptomycin prescription	
Glycopeptides	37 (86)
Fluoroquinolones	27 (63)
Rifampin	25 (58)
Clindamycin, pristinamycin	18 (42)
Piperacillin tazobactam	15 (35)
Cephalosporins	13 (30)
Fosfomycin	13 (30)
Oxacillin, cloxacillin	12 (28)
Aminoglycosids	9 (21)
Fucidic acid	7 (16)
Linezolid	5 (12)
Others	18 (42)

Data are *n* (%) unless otherwise indicated

*ASA* American Society of Anesthesiologists*, BJI* Bone and joint infection*, BMI* Body mass index*, MDRD* Modification of the diet in renal disease

### Microbiology and daptomycin prescription patterns

Culture results from deep samples included 33 bone samples (77 %), 5 joint aspirate samples (12 %), and 2 other deep samples (5 %). Coagulase-negative staphylococcus was implicated in 32 patients (74 %), *S. aureus* in 11 (26 %), *P. acnes* in 8 (19 %), *Corynebacterium* spp. in 4 (9 %) and Enterococcus in 3 (7 %). Five of these patients (12 %) were co-infected with Gram-negative bacilli, and 3 with fungi (8 %). Infection was polymicrobial in 23 cases (59 %). Twenty-seven of the 32 coagulase-negative staphylococci isolates (84 %) were methicillin-resistant; vancomycin MIC was >2 mg/L and equal to 2 mg/L in 10 (31 %) and 12 (39 %) isolates, respectively; and 18/32 (56 %) isolates were resistant to teicoplanin. Daptomycin E-test® results were available in 25 isolates among the methicillin-resistant strains (93 %): all were susceptible to daptomycin, with MIC values ranging from 0.064 to 1 mg/L (mean, 0.33 ± 0.27 mg/L).

In *S. aureus*, methicillin resistance was detected in 1 patient (9 %), with conserved susceptibility to glycopeptides, and daptomycin MIC of 0.125 mg/L.

Daptomycin was administered at doses ranging from 6.5–10 mg/kg/d (mean, 8 ± 0.9 mg/kg/d). Thirteen patients (30 %) received >8 mg/kg/d. Daptomycin was included in anti-Gram-positive combination therapy in 37 patients (86 %), most commonly associated with fosfomycin (*n* = 15, 35 %), rifampin (*n* = 9, 21 %) or clindamycin (*n* = 5, 12 %). Fourteen patients (33 %) received daptomycin treatment as outpatient parenteral antibiotic therapy.

### Safety analysis

**Fig. 1:**
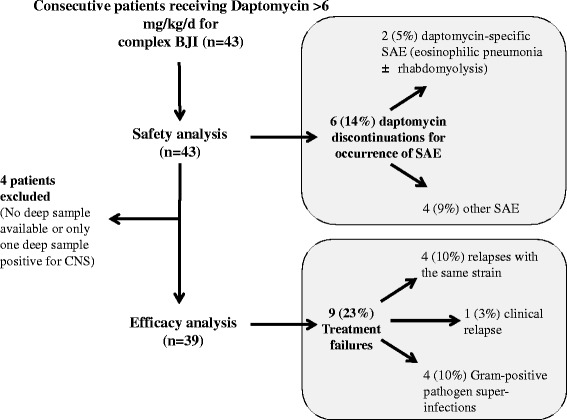
Flow chart of the cohort study. CNS, Coagulase negative staphylococci; SAE, Serious adverse event

**Fig. 2:**
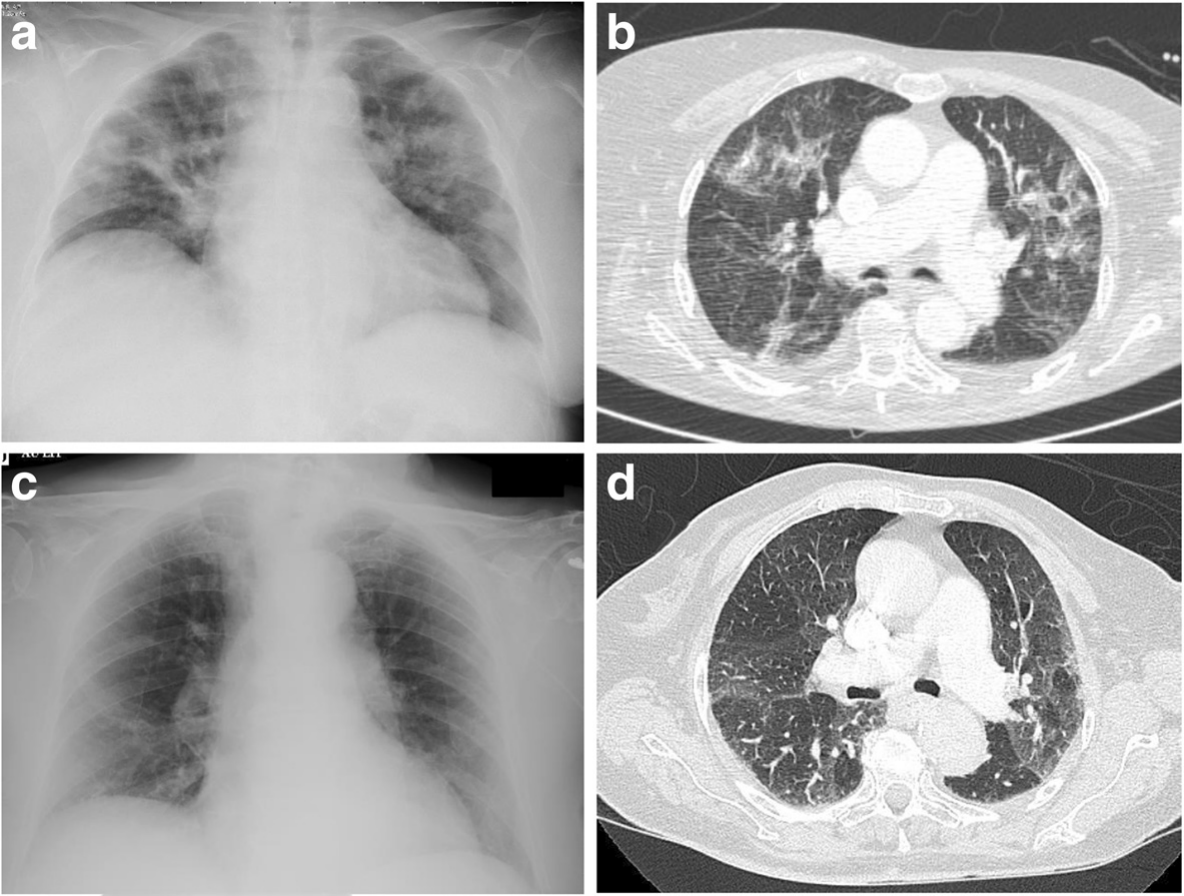
Chest X-ray (**a**, **c**) and computed tomography (**b**, **d**) for the two patients with eosinophilic pneumonia

**Fig. 3:**
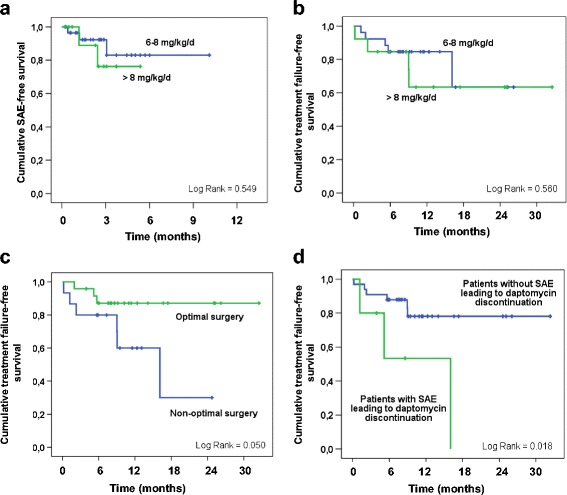
Risk factors for failure and for severe adverse event

The primary reason for stopping daptomycin was completion of therapy (37 patients, 86 %). Mean treatment duration was 81 ± 59 days (range 6–303 days). During daptomycin-based antimicrobial therapy, 17 patients (40 %) experienced 26 AEs (Table 2). Eight patients (16 %) experienced mild AEs, 2 (5 %) moderate AEs and 6 (14 %) SAEs, none of which were life-threatening. AEs occurred after a mean 56 ± 48 days’ daptomycin therapy (range, 6–180 days). All 6 SAEs (Table 3) led to daptomycin withdrawal (Fig. 1). Three patients (7 %) experienced mild transient asymptomatic increase in CK; none were receiving statins. Three patients developed pneumonia, including two cases of certain eosinophilic pneumonia directly implicating daptomycin. These two patients had pulmonary infiltrates and bronchoalveolar fluid with eosinophils (35 % and 11 %, respectively; Fig. 2); of note, both were receiving more than 8 mg/kg/d daptomycin (8.4 and 8.7 mg/kg/d, respectively), and daptomycin C_min_ > 24 mg/L was associated (Table 3). Cox univariate analysis and Kaplan Meier curves showing the cumulative probability of SAEs during daptomycin-based therapy showed no significant differences between patients receiving 6–8 mg/kg/d vs. > 8 mg/kg/d (Fig. 3, panel A). Daptomycin C_min_ was assessed 52 times in 20 patients (47 %) after October 2012; 1 case showed daptomycin C_min_ > 24 mg/L, with asymptomatic elevation of blood CK. Of note, asymptomatic transient daptomycin C_min_ > 24 mg/L was detected in 3 other patients, without elevation of blood CK. C_min_ was not significantly predictive of occurrence of SAE (6 events; HR, 1.092; 95 % CI, 0.950–1.254), whereas it was predictive of occurrence of mild to severe AEs (8 events; HR, 1.037; 95 % CI, 1.002–1.073).

**Table 2:** Adverse events potentially related to the daptomycin-based antimicrobial therapy (*n* = 43) that occurred in 17 of the 43 included patients. Daptomycin dose is expressed in mg/kg/d. AEs onsets are notified in days

Adverse events	*n* (%)	Daptomycin dose (mean, range)	AE onset (mean, range)	Daptomycin withdrawal	Companion drug withdrawal
Hematologic disorders	12 (28)	7,8 (7–9)	44 (7–92)	4/12	3/12
Hypereosinophilia	6 (14)	7,8 (7–9)	43 (10–92)	3/6	1/6
Neutropenia	4 (9)	7,8 (7–9)	73 (49–88)	1/4	2/4
Increased blood CPK	4 (9)	7,5 (7–8)	49 (9–92)	2/4	2/4
PICC thrombosis	3 (7)	8,3 (7–9)	39 (12–79)	1/3	0/3
Hepatic disorders	3 (7)	8,2 (7,5–9)	71 (13–112)	0/3	1/3
Eosinophilic pneumonia	2 (5)	8,5 (8–9)	17 (6–23)	2/2	1/1
Increased blood creatinine	1 (2)	8	8	1	1
Pancreatitis	1 (2)	8	180	0	1

*AE,* Adverse event, *CK*, Creatine kinase, *PICC*, Peripherally inserted central catheter

**Table 3:** Serious adverse events (SAE) occurring in 6 patients under daptomycin-based antimicrobial therapy

Patient	Dose (mg/kg/d)	Additional antibiotic	SAE	SAE onset (days)	C_min_ at SAE onset (mg/L)	Daptomycin withdrawal	Companion drug withdrawal
**1**	8,8	Rifampicin*	Neutropenia	73	NA	Yes	Yes
**2**	7,1	Rifampicin	Hypereosinophilia	92	NA	Yes	No
**3**	8,4	Rifampicin	**Eosinophilic pneumonia, rhabdomyolysis**	**10**	**134**	Yes	Yes
**4**	8,7	Pristinamycin	PICC thrombosis	12	NA	Yes	No
**5**	8,7	None	**Eosinophilic pneumonia**	**23**	**38**	Yes	NA
**6**	7,8	Linezolid	Acute renal failure	8	NA	Yes	Yes

In bold: SAE directly attributed to daptomycin

*NA* Not available, *PICC* Peripherally inserted central catheter, *SAE* Serious adverse event

*Rifampicin can induce both neutropenia and eosinophilia

### Efficacy analysis

Four cases of osteitis were excluded from the efficacy analysis (Fig. 1). Two of the 39 patients included (5 %) underwent no surgery, because of severe underlying disease. Surgery was considered optimal in 24 patients (62 %). During follow-up (387 ± 243 days), treatment failure was recorded for 9 patients (23 %) (Fig. 1), all concerning implant-associated BJI. None of the strains involved developed daptomycin MIC elevation during the study period. All patients in whom daptomycin was discontinued experienced relapse. Univariate Cox analysis and Kaplan-Meier curves showed that dose exceeding 8 mg/kg/day was not associated with better outcome (Table 4; Fig. 3, panel B). Three factors were significantly associated with treatment failure: age (hazard ratio [HR], 1.89; 95 % confidence interval (CI), 1.03–3.47), non-optimal surgery (HR, 3.63, 95 % CI, 0.91–14.73) and SAE leading to daptomycin discontinuation (HR, 4.84; 95 % CI, 1.17–20.05) (Table 4; Fig. 3, panel C and D).

**Table 4:** Cox univariate analysis showing variables associated with treatment failure during daptomycin-based antimicrobial therapy

Variable	*n* (%)	unadjusted HR (95 % CI)	*p*-value
Age (per 10 years)	NA	1.89 (1.03–3.47)	0.041
Male sex	23 (59)	1.48 (0.25–1.48)	0.245
Obesity	12 (31)	1.06 (0.93–1.06)	0.932
ASA score	NA	1.11 (0.79–1.11)	0.787
Smoking	13 (33)	0.91 (0.23–3.65)	0.896
Implant associated BJI	33 (85)	27.8 (0.02–40422.69)	0.371
Chronic BJI	5 (13)	1.15 (0.14–9.22)	0.894
Fistula	14 (36)	2.94 (0.60–14.43)	0.185
Relapsing BJI	15 (63)	5.50 (0.69–44.02)	0.108
*S. aureus*	11 (28)	0.59 (0.12–2.89)	0.517
No or non-optimal surgery	15 (38)	3.63 (0.91–14.73)	0.068
Previous treatment with glycopeptides	34 (87)	25.47 (0.01–142518.48)	0.462
Glycopeptide-resistant isolate	20 (51)	2.965 (0.70–12.58)	0.141
Daptomycin ≤ 8 mg/kg/d	26 (67)	0.676 (0.18–2.55)	0.563
Daptomycin discontinuation for SAE	6 (15)	4.844 (1.17–20.05)	0.029

*ASA* American Society of Anesthesiologists*, BJI* bone and joint infection*, HR*, hazard ratio*, NA* not applicable (continuous variable), *SAE* serious adverse events

## Discussion

Despite its non-comparative, non-randomized, single-center design, this cohort study provides important data concerning off-label use of prolonged high-dose daptomycin therapy in complex BJI, reflecting the real-life use of daptomycin in a BJI referral center. Daptomycin therapy in patients with complex BJI shows a high rate of treatment success (77 %), despite the patient population being at high risk of relapse. Age, non-optimal surgery and daptomycin discontinuation for serious adverse events were the three determinants of treatment failure. Daptomycin was safe, but a higher incidence of eosinophilic pneumonia than expected was recorded, in contexts of daptomycin C_min_ > 24 mg/L.

Daptomycin does not have approval for the treatment of BJI by the US and European authorities. However, based on retrospective data and one randomized controlled trial, it was proposed as an alternative option in patients with staphylococcus or enterococcus prosthetic joint infection in the recent IDSA guidelines, at the standard dose of 6 mg/kg/d [4, 5, 7]. Recent reports of poor daptomycin bone penetration encourage the use of higher doses [6]. In addition to these pharmacological studies, doses up to 8 mg/kg are supported by in vitro [9, 10] and in vivo studies [11–13] High doses have been described in retrospective studies [14–16], but prospective studies including patients receiving high doses of daptomycin are crucial to determine efficacy and safety in BJI.

Concerning safety, patients with BJI are particularly exposed to serious adverse events, as most antimicrobials are used in combination, at high doses and for several weeks or months [3]. In the present study, 40 % of patients experienced at least one AE during treatment, which is notably higher than in previous reports [7]. This difference can probably be explained by longer treatment duration (81 days, versus 28 days in the EU-CORE^SM^ database), as AEs occurred after a mean 56 days. Despite this high AE rate, only 6 patients experienced an SAE that led to daptomycin discontinuation, which is consistent with the 10 % rate of toxicity-related daptomycin withdrawal observed in a recent retrospective series of 20 patients [16]. Of note, 2 of the 6 presented confirmed eosinophilic pneumonia (5 % of patients), which is more than expected, as eosinophilic pneumonia is considered to be an infrequent daptomycin-related SAE: 0.5 % of patients receiving daptomycin in the retrospective post-marketing Eu-CORE® study experienced eosinophilic pneumonia, and a recent review of the literature reported only 24 published cases [17]. The pathophysiology of daptomycin-associated eosinophilic pneumonia is unclear, and it is unknown whether high doses of daptomycin might contribute to the occurrence of eosinophilic pneumonia. In vitro investigations suggest that hydrophilic proteins contained in the pulmonary surfactant (particularly surfactant protein C) bind to daptomycin, inhibit its antibacterial activity and facilitate the formation of surfactant aggregates [18]. The relation between eosinophilic pneumonia and daptomycin C_min_ > 24 mg/L has to be explored, especially as there was daptomycin C_min_ > 24 mg/L in both patients with eosinophilic pneumonia. Daptomycin C_min_ > 24 mg/L in plasma may lead to drug accumulation in the alveolar spaces and induce formation of surfactant aggregates, followed by eosinophil recruitment in lung parenchyma.

Moreover, 6 patients are presented in Table 3 with serious adverse events: 4 of them with dose higher than 8 mg/kg and 2 of them with dose higher than 7 mg/kg. Variables associated with treatment failure did not include daptomycin dose lower than 8 mg/kg: regarding these data, we do not recommend to use doses higher than 8 mg/kg/d.

Concerning efficacy, there was a high success rate (77 %) in a population at high risk of failure, especially as most of our patients presented chronic relapsing implant-associated BJI. This rate is consistent with the 75 % clinical cure rate reported by Seaton et al. [7]. Of note, in the present study: (i) daptomycin was used as second-line antimicrobial therapy in patients often previously exposed to vancomycin; and (ii) half of the patients (22 patients, 51 %) were infected with Gram-positive pathogens with decreased susceptibility to glycopeptides (mostly teicoplanin-resistant coagulase-negative staphylococci, and 10 patients with vancomycin-resistant strains). These two conditions have been reported to facilitate acquisition of daptomycin-resistance during treatment in patients with persistent bacteraemia or endocarditis [19–21]. Of note, in the present series, patients experiencing treatment failure did not show acquired daptomycin-resistance.

In the present study, as in others, age emerged as a risk factor for treatment failure [22]. Interestingly, patients for whom daptomycin was withdrawn for SAE were also at higher risk of treatment failure. This is easily explained, inasmuch as daptomycin was mostly used as salvage therapy, and very few other treatment options were available for our patients. Finally, non-optimal surgery was also a risk factor for relapse, highlighting surgical management as the cornerstone of the treatment of complex BJI. For instance, in patients with chronic orthopaedic implant-associated infection, total removal of the implant is required to limit bacterial persistence [23]. Likewise, it is of the utmost importance to remove the cement in patients with cemented prosthesis joint infection. As removing the cement without inducing bone fracture is usually challenging, particular improved techniques, such as ultrasonic cement removal, are used in reference centers, limiting the need for osteotomy and preserving the bone stock that is essential for reconstruction and reimplantation [24].

Consequently, in case of difficult-to-treat BJI, in which relapse is associated with severe disabilities, the complex choice of aggressive surgical strategies and the difficult monitoring of off-label use of alternate therapies such as daptomycin fully justify management in a regional reference center. The French care network created in 2008 by the French Health Ministry (i.e., management of patients with complex BJI in one of the 9 approved regional reference centers, in collaboration with primary public or private-sector hospitals) may improve prognosis in one of the most difficult-to-treat bacterial infections.

## Conclusion

In conclusion, prolonged high-dose daptomycin was associated with a high success rate in our cohort of patients with complex BJI, partly because antimicrobial therapy was associated with optimal surgery in a regional reference cente, which is essential for the prognosis of complex BJI. Daptomycin was safe, although there was a higher incidence of eosinophilic pneumonia than expected, surprisingly in patients with daptomycin C_min_ > 24 mg/L. Based on our results, 8 mg/kg/d (and no more) seems to be the adequate dose of daptomycin for the treatment of BJI. Monitoring daptomycin levels seems important in patients receiving high-doses of daptomycin, to limit occurrence of adverse events.

## Abbreviations

AE: adverse event
BJI: bone and joint infection
CK: creatine kinase
C_min_: trough plasma concentration
CTCAE: common terminology criteria for adverse events
DGOS: *Direction Générale de l’Offre de Soins*
EUCAST: European Committee on Antimicrobial Susceptibility Testing
FDA: Food and Drug Administration
HPLC: high performance liquid chromatography
IDSA: Infectious Diseases Society of America
MIC: minimum inhibitory concentration
SAE: severe adverse event
